# Mr. Blegborough, on the Air-Pump Vapour Bath

**Published:** 1802-07

**Authors:** Ralph Blegborough

**Affiliations:** Oxford Street, No. 330


### *To the Editors of the Medical and Phyſical Journal.*

GENTLEMEN,

I Had the honor of addreſſing you in February laſt on the ſubject of the Air Pump Vapour Bath, a plate of which I tranſmitted to you. Your attention in having given it a place in the Journal of April, demands my beſt acknowledgements. It was then my intention not to have troubled you again ſo early, but rather to have waited the remarks and obſervations of others; none having yet been publicly made, at the ſuggeſtions of ſome of my medical friends, and from a thorough conviction in my own mind of the importance of the thing, I have been induced to alter my intention, and to attempt ſome further account of it myſelf.

Several medical gentlemen, who called on me in conſequence of my former letter, expreſſed their approbation and ſurprize, particularly on finding the apparatus no larger than it is. One, the phyſician to an hoſpital, ſaid, "Why, 'tis not much larger than a jack boot; really, my friend, you ought to have ſent a ſcale of it, as I, for one, had imagined from the plate, that it bore ſome reſemblance to a brewer's cauldron." This I think neceſſary to remedy by the ſcale* herewith ſent, by which your readers will perceive, that though it is a *little* larger than a jack boot, as that was neceſſary for the circumambient ſteam, yet that it is by no means ſo large as not to be eaſily portable, more eſpecially as the pneumatic parts take off, and can be eaſily placed along with the boiler into the inſide of the cylinder, and the whole encloſed in a neat mahogany box. A board too I had forgotten to mention, on which the leg reſts while in the machine. It conſiſts of one piece as long as the leg, and another in the ſhape of the foot, which goes off at right angles, and is dovetailed to the leg-piece. The whole is ſo contrived, as to defend the limb from the current of ſteam, and to ſuffer it to diffuſe itſelf equably through the machine.

A ſecond gentleman declared there was nothing new in the invention, that it was originally a contrivance of Medea, the

* The depth of that part of the machine to which the pneumatic parts are affixed, 1 foot 2 inches. The other, 1 foot. The length of the cylinder, 2 feet 2 inches. The opening for the admiſſion of the limb, 7 inches by 5½. The cylinder is of an oval ſhape, but ſtands conveniently in a mahogany caſe, which has a flat bottom.

wife of Jason, by which ſhe made her father-in-law Æſon, young again. In proof of his aſſertion he quoted the following paſſages:

"Paſſis Medea capillis

Bacchantum ritu flagrantibus circiter aras;

Multifidas faces in foſſâ ſanguinis atrâ

Tingit, et intinctas geminis accendit in aris.

Terque ſenem flammâ, ter aquâ, ter ſulphure luſtrat.

Interea validum poſito medicamen aheno

Fervet, et exſultat, ſpumiſque tumentibus albet."

"——— Æſon miratur et olim

Ante quater denos hunc ſe reminiſcitur annos."

OVID METAM. Lib. vii. Fab. 2.

The coincidence of many of the leading facts between Mrs. Medea's kettle and ours are certainly very ſtriking, particularly in the power they both poſſeſs of making people young again. There can be no doubt of the fact; and the proof of it is, that they were in all probability arrived at by very different trains of reaſoning, Mr. Smith having never in his life heard of ſuch a perſonage as Mrs. Medea, much leſs of her kettle.

A third gentleman obſerved, that he ſuppoſed it was a machine invented to anſwer ſome preconceived opinion of the inventor. This, Gentlemen, was not the caſe; the inventor is not a medical man, nor had he, at the time of the invention, any thing more in his head than what I thought neceſſary to ſtate in my former letter, viz. the old ſtory of King Edward and Queen Eleanor at the Cruſades. This, however ludicrous it may appear, was what gave riſe to the invention. The above remark, neverthelefs, was to me a moſt unlucky one; for I am free to own, that I had been violently ſeized with the Cacoethes Scribendi. In three months I had produced the moſt ſplendid Hypotheſis reſpecting the Machine you can ſuppoſe! Were I to tell you how ſplendid it is, you might accuſe me *of*, but could not blame me *for*, my partiality to it; for, as Gay ſays,

"Where yet was ever found the mother,
Who'd give her booby for another?"

I am not ſure that the above remark, unlucky as it was, would have been ſufficient to have put me out of conceit with my hypotheſis, had I not alſo recollected what vile purpoſes* I had ſeen thoſe of others put to, which excited in me a kindred ſympathy for the fate of my own. I reviſed it with great attention

* Et piper, et quicquid chartis amicitur ineptis. HORACE.

tion, and muſt own that I did not feel all my former affection for it. At this moment,

> " Cynthius aurem
> Vellit et admonuit."

> " Sunt geminæ ſomni portæ, quarum altera fertur

> Cornæa, quâ veris facilis datur exitus umbris;

> Altera candenti perfecta nitens elephanto;

> Sed falſa ad cœlum mittunt inſomnia manes.

> His ibi tum me

> Proſequitur dictis, portâque emittit eburnâ."

VIRGIL, ÆN. vi. line 898.

Apollo alſo ſuggeſted that I ſhould place my hypotheſis into the machine, and treat it after the manner of a gouty limb. Whimſical as the idea was, it was done. Nothing remarkable happened during the proceſs. Upon examining the cylinder afterwards, in which I expected to find ſome caput mortuum, ſtrange to tell, there was not the leaſt: It had all vaniſhed into thin air, and, like all other hypotheſes, ſeemed to conſiſt of

" Vox et præterea nihil."

This determined me to abandon it altogether and ſtick to facts. But think, gentlemen, what ſuch a determination muſt have coſt me, who, not many days before, had been hugging myſelf with the idea that I had produced an hypotheſis (for I call none theories till facts have proved them ſo) which was to aſtoniſh the world, and laſt for ages!* And now, gentlemen, any of your readers may retire by the ivory gate; thoſe who will patiently hear me out, I promiſe to conduct ſafely through that of horn. So much for hypotheſis! and now to be very ſerious. Since I laſt wrote to you I have witneſſed ſome very extraordinary effects from the application of the Air-Pump Vapour Bath, ſuch as from an extenſive practice in all the branches of the medical department for the laſt ten years, I am convinced could not have been produced by any other means with which we are already acquainted.

I ſhall now ſtate a few caſes† out of numbers which have occurred to me lately, and ſhall chiefly make a point of ſelecting thoſe of ſuch perſons as are leaſt difficult of acceſs; and firſt, that of Mr. Seares, the ſurgeon, of Half-Moon Street, Piccadilly, to whom I am indebted for the following ſtatement, which I ſhall give in his own words.

ACUTE

---

* " Exegi monumentum ære perennius." HORACE.

† " Segnius irritant animos demiſſa per aurem

Quam quæ ſunt oculis ſubjecta fidelibus." HORACE.

### Acute Gout.

"Dear Sir, *Half-Moon Street, May* 31, 1802.

"Seeing in the Medical and Phyfical Journal, a letter of yours refpecting a machine for conveying a vapour bath to difeafed limbs, I could not poffibly think of withholding my teftimony of its beneficial effects in gout; I can fpeak of it feelingly and with gratitude, having experienced fuch relief from it myfelf in a moft violent attack of gout in the extremities.

In the winter of 1799, by being thrown out off my chaife, I received a violent contufion on the left foot, which terminated in a moft fevere paroxyfm of the gout; it confined me a confiderable time, and rendered me unable to purfue my profeffional avocations; the dread of a future attack may be readily conceived. The following winter confirmed my fears, and I was vifited by another paroxyfm with increafed violence, for great as my fufferings were before, they were trifling indeed compared with what I fuffered then. Fortunately, by mere chance, I heard that a gentleman at Pimlico, whom I knew, had received the greateft benefit from the application of the machine; I fent therefore to Mr. James, the proprietor of it, to hear from him the principles of its operation; and as it appeared to me to be perfectly innocent, I was willing to embrace fuch a mode as would be likely to relieve my pain, and enable me more fpeedily to profecute my profeffional duties. It fucceeded beyond my moft fanguine expectations, as on the evening previous to its application I could not bear my feet to touch the ground; after the machine had been applied, I could prefs on them without pain. The fucceeding application was ftill more gratifying, as I could fhortly afterwards walk with very little affiftance about the room; and the next day I fuffered only from ftiffnefs and the fear of reproducing an attack by any blow I might receive on the parts. This however fubfided, and I was enabled in a day or two to attend to bufinefs. Grateful for the benefit I have received, I could not withhold giving you this hafty fketch. Without apologizing for its imperfections, I hope this, with other documents, will tend to make the machine more generally known, and I truft approved.

"I am, dear Sir,

"Your's, obediently,

"G. H. Seares."

"*To Mr. Blegborough*, 330, *Oxford Street.*"

After the above ftatement of Mr. Seares, little further is neceffary to be faid on the acute part of the difeafe, I fhall therefore but mention one other cafe.

A Mr.

A Mr. Smith, of 47, Oxford Street, on the 20th of March, was labouring under a moft fevere paroxyfm. He has been fubject to frequent returns of its attacks, which always continue with him fome time. The application was made on the 20th and 22d, under fome unfavourable circumftances and irregularities in improperly moving. Neverthelefs, on the 23d and 25th it was applied again under more favourable circumftances. The paroxyfm entirely remitted, fince which time he has continued very well; he efcaped all the fubfequent debility, which never failed to fucceed former attacks, that were fuffered to run their courfe under patience and flannel; and got into his bufinefs immediately. I hope I have given him fuch directions refpecting his future regimen as will keep him out of the machine for a great length of time to come. It may not be amifs to remark here, that this patient confiders the application, independent of its utility, as a luxury in no inconfiderable degree exquifite.

### Irregular Gout.

I am happy in having rendered Mr. Mortlock, of Cambridge, much fervice in a complaint which comes under the above defcription. There feemed a ftrong general gouty diathefis; his ftomach was much affected, into which he was putting a quantity of ginger every morning, a practice I could by no means approve, though much extolled by a very worthy Baronet. I gave him fome general directions refpecting regimen, and enforced the ufe of milk. The machine was applied fifteen times to his leg and arm. It feemed firft to determine the affection to thefe two points, and foon to remove it altogether. He left town much improved in general health as well as nearly cured of the particular affection. I went to make him a vifit at Abington near Cambridge a fortnight after; a confiderable thickening and hardnefs of the ancle had given way, and he was much better in general health. He gave me letters to fome of his friends, one of which I fhall take the liberty with him of tranfcribing.

"*Abington Hall, April* 10, 1802.

"Dear Sir,

"I know not whether you are ever afflicted with the gout; but knowing it has been feverely felt in your family, I have requefted my friend Mr. Blegborough, a furgeon in London, who is proprietor of a machine, which has done me very effential benefit, both for regular and irregular fits of the complaint. I will thank you to introduce him to your furgeon, not only as he wifhes to make the inftrument known, but alfo, as you will by him be affured that it can do no harm. It would give give me great pleafure to fee you here; but I have long found fo great a difficulty of detaining you when I was at Cambridge, and you there, that I now quite defpair of it. I am,

"Dear Sir,

"Your's, moft truly,

"JOHN MORTLOCK."

"*To James Denton, Esq. Brandon.*"

ACUTE RHEUMATISM.

On the 20th of January, 1802, Mr. Clarke, of No. 18, Somerfet Street, Portman Square, had been feverely afflicted with Rheumatifm for many months, fo as not to be able to move without the moft excruciating pain. It affected his breaft, fhoulders, and joints, and the mufcles about the breaft, that he was not able to breathe without confiderable difficulty, nor could he by any means ftoop to take any thing from the ground, or bring himfelf perpendicular, but was under the neceffity of inclining forward at an angle of about 135 deg. On the above date I firft faw him; he had been under the care of regular men, and all ordinary means had been ufed without effect. He had been to Bath, and nothing feemed to have been omitted which promifed relief. His pulfe was quick but fmall, tongue white, fkin dry, parched, and hard, confiderable thirft, and no appetite.

I directed him to take half an ounce of Epfom's falts with half a drachm of magnefia in two ounces of peppermint water, and took ten ounces of blood from the arm, and ordered the application of the machine the following day. On the 22d in the morning I found him much relieved. The blood fhewed the inflammatory buff. There feemed a much greater freedom of circulation, and the pulfe was more full and not fo frequent; but the moft remarkable change had taken place on the fkin, which had become foft, moift, and perfpirable. The patient could turn in bed, and the latitude of motion in other refpects was much more confiderable. The machine was applied on the evening of the 22d, and again on the 24th, on which day I ordered eight ounces more blood to be taken from the arm. The operation was mifmanaged, and only two ounces were procured. On this day he repeated his draught, and on the following was fo well as to induce me to inform him that he need ufe the machine no more; as he could now bend the body almoft as freely as natural, and was entirely free from pain. He, however, of his own accord, chofe to have another application, which was made on the 27th; fince which time he has remained remarkably well.

I fhall ftate another cafe, as bleeding was employed in the above above, though to a very fmall amount; yet it may, on that account, be objected to by fome as not a fair one.

Thomas Pearce, a poor bricklayer's boy, Ward's Fields, New Road, Mary-le-Bone, on the 13th of May, inftant, had been confined to bed for eight weeks with the difeafe. I faw him at the requeft of a medical gentleman. He was unable to ftraight his knees, which were much contracted and enlarged. His legs and thighs were literally worn to the bone; and fuch was the general debility, that it left little hope of its being poffible that he fhould have fprung from the difeafe by any ordinary means, which had all been duly adminiftered. His left hand and arm had much the appearance of a paralytic limb, except round the joints, which were fwelled, and were as ufelefs. The application was firft made to the left leg twice with confiderable relief; then to the left arm twice; then the right leg and arm, each once. His loft appetite began to return in proportion as the pain, &c. fubfided. I found it neceffary to regulate the porter he drank; but left him at liberty to eat any animal food they could procure for him. He gradually recovered, and has been, on the 28th, to the houfes of two medical gentlemen to return them thanks; the one for recommending the machine to him, the other for his care and humanity towards him prior to the ufe of it.

I have feldom met in practice with a circumftance more pleafant to my feelings than the above cafe; that I recovered to this poor boy the ufe of his limbs, and to fociety a ufeful member, otherwife in all probability loft for ever, I firmly believe; nor fhall the united fcepticifm of the world perfuade me to the contrary.

### Palsy.

John Rouneberg, No. 11, James Street, Brook Street, a patient of the Mary-le-Bone Difpenfary, who, after being under the care of Dr. Thornton, the late phyfician, and ufing among other things, the vital air without benefit, was fent to me by Dr. Garnett. He has been paralytic for two years, which led me to expect very little from the machine; neverthelefs, at the requeft of the Doctor it was tried. The effect has been very confiderable, for after fix applications he has much more ufe of his arm, fo that he can now lift it over his head without the help of the other hand, can grafp any thing firmly, and carry a large pitcher of water, which he could by no means do before. A general ftate of excitement, however, has pervaded the whole fyftem, fo that I have deemed it prudent to defift from the application for the prefent. This general excitement, I have little doubt, was produced from the local one of the arm by the increafed circulation and capacity of the veffels of the part. As foon as it fhall have fubfided, which is nearly nearly the cafe, I fhall proceed, keeping in view *feftina lenté*, and hope I fhall be enabled hereafter to give a ftill better account of a cafe, which at this early period of it, I could not have omitted without calling in queftion the judgment of Dr. Garnett, to which I fhall ever bow with deference.

Derangement of the Functions of the Bladder.

Capt. Rufh, No. 25, Nottingham Place, New Road, Mary-le-Bone, was relieved from a moft violent affection of the bladder of long ftanding. The conftans mingendi cupido was fo fevere that he declared life was not worth the tenure. From the quantity of mucus difcharged, the inner coat of the bladder muft have been entirely abraded. It would be prefumptuous in me to hazard an opinion refpecting the caufes, as various ones had been given by a number of gentlemen who had been confulted; ftone and gout among the reft. The affection, however, entirely ceafed after a few applications of the machine, a trial of which Dr. Frazer, who was attending at the time, had no objection to.

*Farthingo, near Brackley, Northamptonfhire, June 5, 1802.*

"Dear Sir,

"It is with much pleafure I inform you of the continuance of the great benefit I received from the application of your air-pump vapour bath. To have given fo much relief in fo fhort a time, and that in a cafe the moft unfortunate that ever man was afflicted with, a cafe that the firft affiftance could fcarce give me any relief from, certainly difcovers a power from which, under your direction, every good may be expected. It has been recommended and introduced as giving relief in gouty cafes; but although, as in my cafe, the gout formed a part of my complaint, yet be affured, Sir, that the other part of my complaint (inflammation of the neck of the bladder) was the caufe from which my great fufferings proceeded.

As my phyfician, Dr. Frazer, who is one of the moft liberal of men, attended me during the application of your remedy, and to whom I explained its effects in progreffion, I cannot do better than refer you to his teftimony. I cannot omit acknowledging the candid and polite manner in which you pointed out the attention you thought neceffary during the courfe of my application, a circumftance much to be defired by any one making the trial. Accept, Sir, every good wifh from

"Your very obedient fervant,

"George Rush."

The lady of a defervedly eminent phyfician in town, and a furgeon a furgeon of great refpectability, are at prefent ufing the apparatus by the recommendation of Mr. Cline.

To mention even the names of all the the phyficians and furgeons in town and country, who have expreffed their approbation of it, would, after what has been faid, but ufelefsly fwell this letter, already too long.

Judging from the number of correfpondents that my former letter added to my lift, I think it neceffary to requeft, that thofe gentlemen from the country, who wifh to have further information refpecting it, will have the goodnefs to direct (poft paid) to Mr. James, one of the Patentees, No. 5, Cumberland Street, New Road, Mary-le-Bone.

I remain, &c.

*Oxford Street, No.* 330, RALPH BLEGBOROUGH.

*June* 7, 1802.

